# Molecular correlates and prognostic significance of SATB1 expression in colorectal cancer

**DOI:** 10.1186/1746-1596-7-115

**Published:** 2012-08-30

**Authors:** Björn Nodin, Henrik Johannesson, Sakarias Wangefjord, Darran P O’Connor, Kajsa Ericson Lindquist, Mathias Uhlén, Karin Jirström, Jakob Eberhard

**Affiliations:** 1 Department of Clinical Sciences, Pathology, Lund University, Skåne University Hospital, Lund, SE, 221 85, Sweden; 2 Atlas Antibodies AB, AlbaNova University Center, Stockholm, SE, 106 91, Sweden; 3 UCD School of Biomolecular and Biomedical Science, UCD Conway Institute, University College Dublin, Dublin 4, Belfield, Ireland; 4 Science for Life Laboratory, AlbaNova University Center, Royal Institute of Technology, Stockholm, Sweden; 5 School of Biotechnology, AlbaNova University Center, Royal Institute of Technology, Stockholm, Sweden; 6 Department of Clinical Sciences, Oncology, Lund University, Skåne University Hospital, Lund, SE, 221 85, Sweden

## Abstract

**Background:**

Special AT-rich sequence-binding protein 1 (SATB1) is a global gene regulator that has been reported to confer malignant behavior and associate with poor prognosis in several cancer forms. SATB1 expression has been demonstrated to correlate with unfavourable tumour characteristics in rectal cancer, but its association with clinical outcome in colorectal cancer (CRC) remains unclear. In this study, we examined the prognostic impact of SATB1 expression in CRC, and its association with important molecular characteristics; i.e. beta-catenin overexpression, microsatellite instability (MSI) screening status, and SATB2 expression.

**Methods:**

Immunohistochemical expression of SATB1 and beta-catenin was assessed in tissue microarrays with tumours from 529 incident CRC cases in the prospective population-based Malmö Diet and Cancer Study, previously analysed for SATB2 expression and MSI screening status. Spearmans Rho and Chi-Square tests were used to explore correlations between SATB1 expression, clinicopathological and investigative parameters. Kaplan Meier analysis and Cox proportional hazards modelling were used to explore the impact of SATB1 expression on cancer specific survival (CSS) and overall survival (OS).

**Results:**

SATB1 was expressed in 222 (42%) CRC cases and negative, or sparsely expressed, in adjacent colorectal mucosa (n = 16). SATB1 expression was significantly associated with microsatellite stable tumours (p < 0.001), beta-catenin overexpression (p < 0.001) and SATB2 expression (p < 0.001). While not prognostic in the full cohort, SATB1 expression was significantly associated with poor prognosis in SATB2 negative tumours (HR = 2.63; 95% CI 1.46-4.71; p_interaction_ = 0.011 for CSS and HR = 2.31; 95% CI 1.32-4.04; p_interaction_ = 0.015 for OS), remaining significant in multivariable analysis.

**Conclusions:**

The results of this study demonstrate that SATB1 expression in CRC is significantly associated with beta-catenin overexpression, microsatellite stability and SATB2 expression. Furthermore, SATB1 expression is a factor of poor prognosis in SATB2 negative tumours. Altogether, these data indicate an important role for SATB1 in colorectal carcinogenesis and suggest prognostically antagonistic effects of SATB1 and SATB2. The mechanistic basis for these observations warrants further study.

**Virtual slides:**

The virtual slide(s) for this article can be found here: http://www.diagnosticpathology.diagnomx.eu/vs/1922643082772076

## Background

Colorectal cancer (CRC) is one of the most common forms of human cancer worldwide with approximately 1 million new cases detected every year [1]. Currently, tumour stage at diagnosis is the most important prognostic factor in CRC and although many efforts have been made to find molecular markers to identify high-risk disease and to select patients for adjuvant treatment, none have proven good enough for use in clinical routine.

We have previously demonstrated that special AT-rich sequence-binding protein 2 (SATB2), a nuclear matrix associated protein and epigenetic regulator that orchestrates the function of multiple genes [2], is expressed in a highly tissue-specific manner in normal mucosa of the lower gastrointestinal tract and in CRC [3,4]. Moreover, loss of SATB2 expression has been shown to correlate with poor prognosis in CRC [4,5].

The T-lineage enriched global chromatin organizer SATB1 [6,7] is a close homologue to SATB2, and expression of SATB1 has been reported to correlate with poor prognosis in several cancer forms, e.g. breast, gastric and liver cancer [8-11]. In a recent study, mRNA and protein levels of SATB1 were found to correlate with unfavourable tumour characteristics in rectal cancer, but the prognostic significance of SATB1 expression was not reported [12]. This study included 93 patients and SATB1 was found to be up-regulated in invasive cancer compared to normal rectal mucosa, but overexpression or positive staining was denoted in < 50% of the tumours, indicating that SATB1 is less abundantly expressed than SATB2 in the lower gastrointestinal tract but may play an important role in colorectal carcinogenesis [12]. SATB2 has also been found to inhibit the expression of SATB1 in human CRC cells in vitro [5]. Moreover, as activation of the WNT signaling pathway and its major mediator beta-catenin is a critical event in colorectal carcinogenesis [13], and SATB1 has been shown to interact with and recruit beta-catenin to its genomic binding sites, the role of SATB1 in CRC development and progression merits further investigation.

The aim of this study was therefore to examine the extent and prognostic significance of SATB1 expression in a large, prospective CRC cohort [14,15]. In addition, we analysed the molecular correlates of SATB1 expression with beta-catenin overexpression, MSI screening status and SATB2 expression.

## Methods

### Study group

Until end of follow-up 31 December 2008, 626 incident cases of CRC had been registered in the prospective, population-based cohort study Malmö Diet and Cancer Study (MDCS) [16]. Cases were identified from the Swedish Cancer Registry up until 31 Dec 2007, and from The Southern Swedish Regional Tumour Registry for the period of 1 Jan - 31 Dec 2008. All tumours with available slides or paraffin blocks were histopathologically re-evaluated on haematoxylin and eosin stained slides. Histopathological, clinical and treatment data were obtained from the clinical and/or pathology records. TNM staging was performed according to the American Joint Committee on Cancer (AJCC). Information on vital status and cause of death was obtained from the Swedish Cause of Death Registry up until 31 Dec 2009. Follow-up started at date of diagnosis and ended at death, emigration or 31 Dec 2009, whichever came first. Median follow-up time was 3.35 years (range 0–17.69) for the full cohort (n = 626) and 6.05 years (range 1.03-17.69) for patients alive (n = 344). Patient and tumour characteristics of the cohort have been described in detail previously [4,14,15]. Ethical permission was obtained from the Ethics Committee at Lund University for the MDCS (Ref. 51/90), and the present study (Ref. 530/2008).

### Tissue microarray construction

Cases with an insufficient amount of tumour material were excluded, whereby a total number of 557 (89.0%) tumours were suitable for tissue microarray (TMA) construction. Areas representative of cancer were marked on haematoxylin & eosin stained slides and TMAs were constructed as previously described [17]. In brief, two 1.0 mm cores were taken from each tumour and mounted in a new recipient block using a semi-automated arraying device (TMArrayer, Pathology Devices, Westminster, MD, USA). As demonstrated previously, there was no selection bias regarding the distribution of clinicopathological characteristics between the TMA cohort and the full cohort [14].

### Immunohistochemistry and staining evaluation

For immunohistochemical analysis, 4 μm TMA-sections were automatically pre-treated using the PT-link system (DAKO, Glostrup, Denmark) and then stained in an Autostainer Plus (DAKO, Glostrup, Denmark). Immunohistochemical staining for SATB1 was performed using a monoclonal anti-SATB1 antibody (Clone EPR3895, Epitomics, Burlingame, CA, USA) diluted 1:100. The estimated fraction of cells with nuclear SATB1 expression was denoted as 0 (0-1%), 1 (2-25%), 2 (26-50%), 3 (51-75%) and 4 (>75%). Nuclear intensity was denoted as negative, weak, moderate or strong, with corresponding scores from 0–3, referring to the predominant intensity. A combined nuclear score (NS) was constructed by multiplying fraction and intensity. SATB1 expression was also evaluated in samples of normal colorectal mucosa from the surgical resection margins (n = 20). Stromal lymphocytes served as positive internal controls.

Immunohistochemical MSI screening status was assessed as previously described [4,18], whereby tumour samples lacking nuclear staining of MLH1, PMS2, MSH2 or MSH6 were considered to have a positive MSI screening status. Hereafter, tumours with a positive MSI screening status are referred to as MSI and tumours with negative MSI screening status are referred to as MSS.

Immunohistochemical staining of beta-catenin was performed with a monoclonal anti-beta-catenin antibody (# 610153 BD Pharmingen, San Diego, Ca, USA), diluted 1:5000. The staining was evaluated as previously described [19] whereby membranous staining was denoted as 0 (present) or 1 (absent), cytoplasmic staining intensity as 0–2 and nuclear staining intensity as 0–2. The total score ranging from 0 (corresponding to membrane staining only, as in normal colonic mucosa) to 5 (tumours with strong nuclear and cytoplasmic staining) was then divided into three categories; 1 = score 0–1, 2 = score 2–3 and 3 = score 4–5). Sample IHC images representing different beta-catenin grades are shown in Additional file 1.

The immunohistochemical stainings was evaluated by two independent observers (BN and KJ), who were blinded to clinical and outcome data. Scoring differences were discussed in order to reach consensus.

### Antibody validation by ELISA and western blot analysis

Since SATB1 and SATB2 are very similar in sequence, the specificity of the anti-SATB1 antibody was analysed using ELISA and Western blot against purified recombinant SATB1 and SATB2 proteins. ELISA-plates (Costar) were coated with purified recombinant MYK/DDK-SATB1 produced in HEK293 cells (#TP300421, Origene Technologies, Rockville, MD, USA) or full-length SATB2 recombinant protein (#H00023314-P01, Abnova, Taiwan) at 1 μg/ml per well and incubated overnight at 4°C. Plates were blocked using 3% BSA in PBS for 1 hr at RT. Anti-SATB1 antibody (Clone EPR3895, Epitomics, Burlingame, CA, USA) was diluted 1:500 in 1% BSA in PBS and added to the wells. Following incubation for 1 hr at RT, plates were washed 3 times with wash buffer (1xPBS/0,05% Tween 20) and an HRP conjugated secondary antibody (#P0399, DakoCytomation) diluted to 1:2000 was added and incubated for 1 hour at RT. Plates were washed as before, substrate (Ready-to-use ABTS solution, Sigma Aldrich, #A3219) was added and allowed to develop for 30 minutes at RT. OD 405 was read on a microtiter plate reader.

For Western blot, 50 ng of purified recombinant MYK/DDK-SATB1 produced in HEK293 cells (#TP300421, Origene Technologies, Rockville, MD, USA) or 50 ng of a full-length SATB2 recombinant protein (#H00023314-P01, Abnova, Taiwan) were separated on precast 4–20% CriterionTGX SDS- PAGE gradient gels (Bio-Rad Laboratories, Hercules, CA) under reducing conditions, followed by blotting to PVDF membranes (Immobilon-P, Millipore), according to the manufacturer's recommendations. Membranes were blocked (5% dry milk, 0.5% Tween20, 1 × TBS) for 45 min at RT prior to addition of antibody (anti-SATB1, Clone EPR3895, Epitomics, Burlingame, CA, USA, diluted 1:200; anti-SATB2, HPA029543, Atlas Antibodies AB, Stockholm, Sweden, diluted 1:250). Following incubation for 1 h with the primary antibody, the membranes were washed 4x5 min in 1xTBS with 0.1% Tween20. An HRP-conjugated swine anti-rabbit antibody (#P0399, DakoCytomation), diluted 1/3000 in blocking buffer, was added to the membrane and incubated for 1 h followed by a final round of washing. Detection was carried out using Chemiluminescence HRP Substrate (Immobilon) according to the manufacturer's instructions.

As demonstrated in Additional file 2 A and B, the anti-SATB1 antibody binds specifically to SATB1 with no discernable cross-reactivity to SATB2. Thus, the antibody is highly specific to SATB1 and does not cross-react to SATB2, despite the extensive sequence similarity shared by the two proteins.

### Statistical analysis

Chi Square and Spearman’s correlation (R) tests were used to explore the associations between SATB1 expression and clinicopathological and investigative parameters. Kaplan-Meier analysis and log rank test were used to illustrate differences in cancer specific survival (CSS) and overall survival (OS) according to three categories of SATB1 expression; negative (NS = 0), intermediate (NS 1–3) and high expression (NS >3), the latter including all tumours with >75% positive nuclei or various fractions of moderate-strong SATB1 staining. Cox regression proportional hazards models were used for estimation of hazard ratios (HRs) for death from CRC or overall causes according to negative and positive SATB1 expression in both uni- and multivariable analysis, adjusted for age (>/<=75 years), gender, T-stage (I-II, III, IV), N-stage (0,1,2), M-stage (0, 1), and differentiation grade (high-intermediate vs low). A backward conditional selection method was used for variable selection by the model. The interaction between SATB1 expression and SATB2 expression was explored by a Cox proportional hazards model including SATB1, SATB2 and an interaction variable. All tests were two-sided. A p-value of ≤ 0.05 was considered significant. All statistical analyses were performed using SPSS Statistics version 18 (SPSS Inc, Chicago, IL).

## Results

### Immunohistochemical expression of SATB1 in colorectal cancer

**Figure 1:**
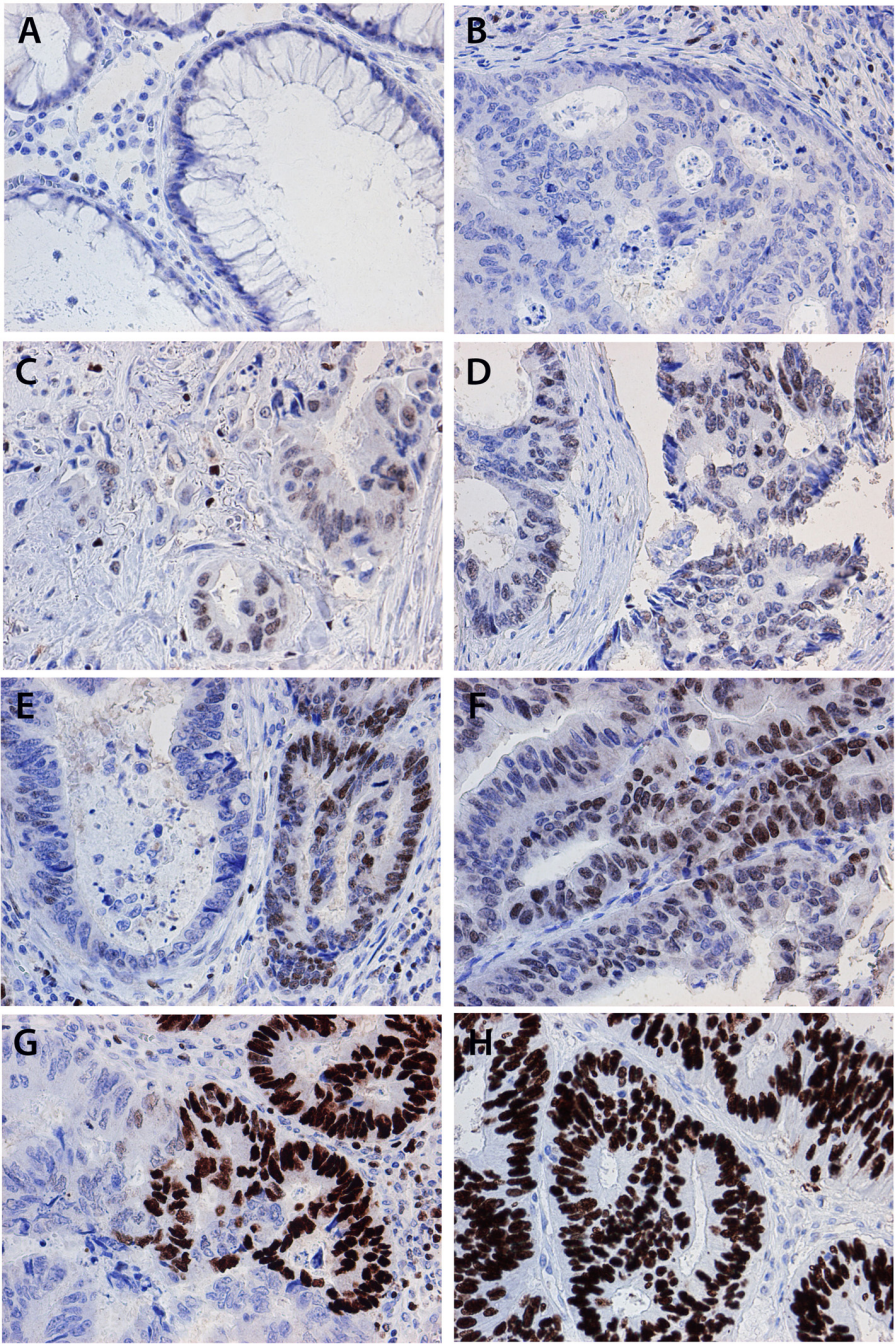
**Immunohistochemical images of SATB1 staining in colorectal cancer and adjacent colorectal mucosa.** Images (20X ) representing immunohistochemical expression of SATB1 staining in (**A**) normal colorectal mucosa and colorectal cancer, ranging from from (**B**) negative through (**C**-**D**) weak intensity, (**E**-**F**) moderate intensity and (**G**-**H**) strong intensity in various fractions.

**Figure 2:**
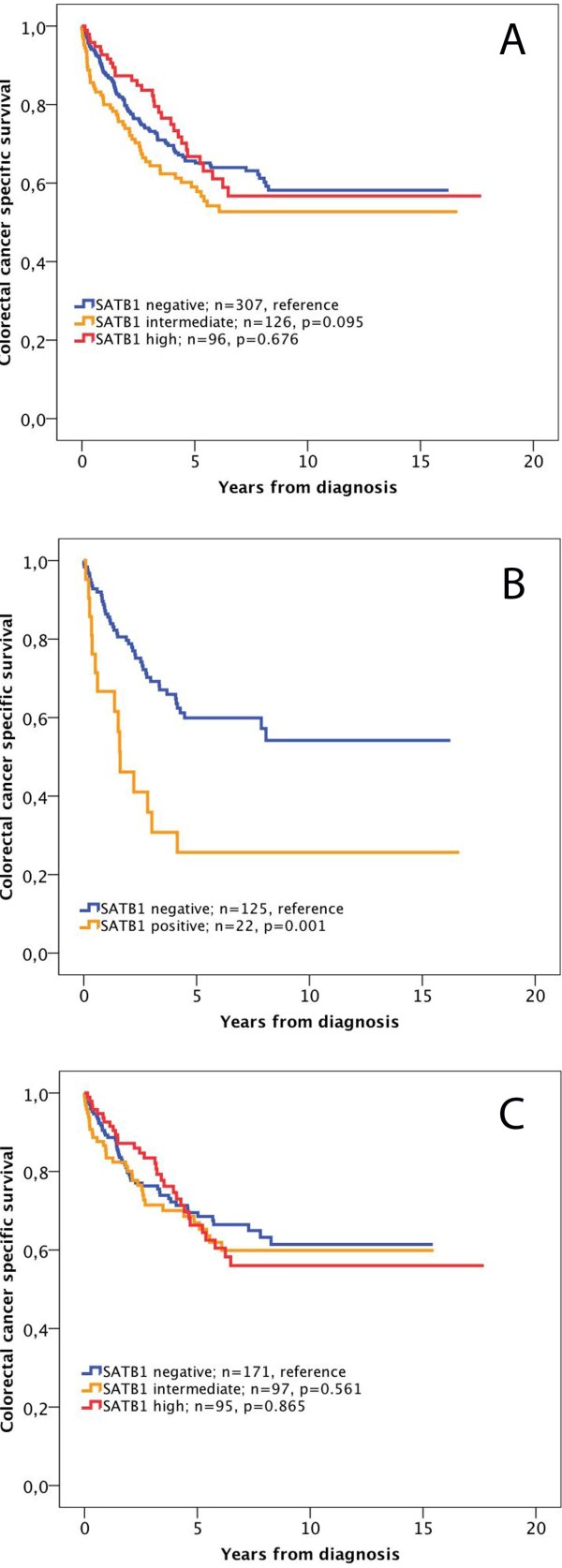
**Kaplan-Meier estimates of the prognostic impact of SATB1 expression in all patients, and patients with SATB2 negative and positive tumours.** Kaplan Meier analysis and log rank test of colorectal cancer specific survival according to negative, intermediate and high SATB1 expression in (**A**) all patients, (**B**) patients with SATB2 negative and (**C**) patients with SATB2 positive tumours. The categories of staining were determined according to the nuclear score (NS), e.g. multiplier of fraction and intensity, whereby negative SATB1 expression = NS 0, intermediate expression = NS 1–3 and strong expression = NS >3.

Following antibody optimisation and staining, SATB1 expression was successfully annotated in 529 (95.0%) tumours and beta-catenin in 527(94.6%) tumours. Sample images of immunohistochemical SATB1 staining are shown in Figure 1. SATB1 expression could be evaluated in 16/20 (80%) samples of adjacent, benign-appearing colorectal mucosa, of which 14 (87.5%) were denoted as having negative expression (Figure 1A) and 2 (2.5%) displayed weak expression in < 10% of cells. In the evaluated CRC cohort, 307 (58.0%) of the tumours were SATB1 negative (Figure 1B), and in the remaining tumours, SATB1 was expressed in various fractions and intensities (Additional file 3). In a small subset of tumours, strong SATB1 expression was seen in the vast majority of tumour cells (NS = 12; n = 18, Figure 1H). Notably, SATB2 was also abundantly expressed in all these tumours, with a NS = 12 in 15/18 cases and a NS = 8 in 3 cases. There was no marked heterogeneity in SATB1 expression between duplicate cores.

### Association between SATB1 expression, clinicopathological and molecular characteristics

Next, we examined the relationship between SATB1 expression and established clinicopathological and investigative parameters. Three categories of staining were compared, i.e. SATB1 negative tumours (NS = 0), an intermediate group (NS = 1-3), and SATB1 high tumours (NS > 3). In the full cohort, SATB1 expression was significantly associated with female gender (p = 0.021), but not with any other conventional clinicopathological parameters (Table 1). A strong, signficant association was however seen between SATB1 expression and MSS tumours (p = <0.001), beta-catenin overexpression (p = <0.001) and SATB2 expression (p = <0.001). In light of the significant correlation between SATB1 and beta-catenin overexpression, we also assessed the association of SATB2 expression with beta-catenin grades, using the same categories as in Table 1. In line with the findings for SATB1, this revealed a strong positive correlation between SATB2 expression and beta-catenin overexpression (p < 0.001). Similar correlations of SATB1 expression with clinicopathological and molecular parameters were seen when the full range of nuclear scores was used in the analyses (data not shown).

**Table 1:** Associations between SATB1 expression, clinicopathological and molecular parameters in all patients, and patients with colon and rectal cancer

**SATB1 Expression**	**Negative**	**Intermediate**	**High**	
n(%)	307(58.0)	126(23.8)	96(18.1)	*p-value§*
*(R)*				
**Age**				
<=75	208(67.8)	86(68.3)	67(69.8)	0.736
>75	99(32.2)	40(31.7)	29(30.2)	(−0.011)
**Gender**				
Female	152(49.5)	71(56.3)	60(62.5)	0.020*
Male	155(50.5)	55(43.7)	36(37.5)	(−0.097)
**T-stage**				
1-2	54(18.2)	30(26.1)	24(25.8)	0.350
3	200(67.3)	67(58.3)	52(55.9)	(−0.042)
4	43(14.5)	18(15.7)	17(18.3)	
*missing*	*10*	*11*	*3*	
**N-Stage**				
0	165(58.1	64(59.8)	55(61.8)	0.719
1	74(26.1)	19(17.8)	21(23.6)	(−0.007)
2	45(15.8)	24(22.4)	13(14.6)	
*missing*	*23*	*19*	*7*	
**M-stage**				
0	252(83.4)	92(74.2)	85(89.5)	0.977
1	50(16.6)	32(25.8)	10(10.5)	(0.036)
*missing*	*5*	*2*	*1*	
**Differentiation grade**				
Intermediate-High	233(77.2)	99(80.5)	70(75.3)	0.964
Low	69(22.8)	24(19.5)	23(24.7)	(−0.009)
*missing*	*5*	*3*	*3*	
**Vascular invasion**				
No	89(47.8)	30(46.2)	29(52.7)	0.696
Yes	97(52.2)	35(53.8)	26(47.3)	(−0.013)
*missing*	*121*	*61*	*41*	
**MSI screening status**				
MSS	227(78.3)	111(94.9)	87(92.6)	<0.001**
MSI	63(21.7)	6(5.1)	7(7.4)	(−0.212)
*missing*	*17*	*11*	*2*	
**Betacatenin grade**				
0	99(33.2	37(30.8)	16(16.8)	<0.001**
1	105(35.2)	35(29.2)	26(27.4)	(0.154)
2	94(31.5)	48(40.0)	53(55.8)	
*missing*	*9*	*6*	*1*	
**SATB2 expression**				
negative	125(42.2)	22(11.2)	0(0.0)	<0.001**
intermediate	133(44.9)	76(63.9)	41(43.2)	(0.392)
high	38(12.8)	21(17.6)	54(56.8)	
*missing*	*11*	*7*	*1*	

Category denoted as negative refers to tumours with SATB1 nuclear score (NS) = 0, intermediate to NS 1–3 and strong to NS > 3. *§* Chi square test for linear trend. R = Spearmans correlation coefficient.The categories marked as not done and unknown were not included in the analysis. *Significant at the 0.05 level. ** Significant at the 0.01 level. N1 = 1-3 positive nodes, N2= > 4 positive nodes. MSS = Microsatellite stable, MSI = Microsatellite unstable. Beta-catenin grade: 0 = score 0–1, 1 = score 2–3, 2 = score 4–5.

### Associations between SATB1 expression and survival

Next, we examined the prognostic impact of SATB1 expression in strata of negative, intermediate and high SATB1 expression (Figure 2). Kaplan Meier analysis revealed no prognostic significance of SATB1 expression for CSS, neither in the full cohort (Figure 2A), nor in separate analyses for colon and rectal cancers (data not shown). Similar findings were seen for OS (data not shown).

**Figure 3:**
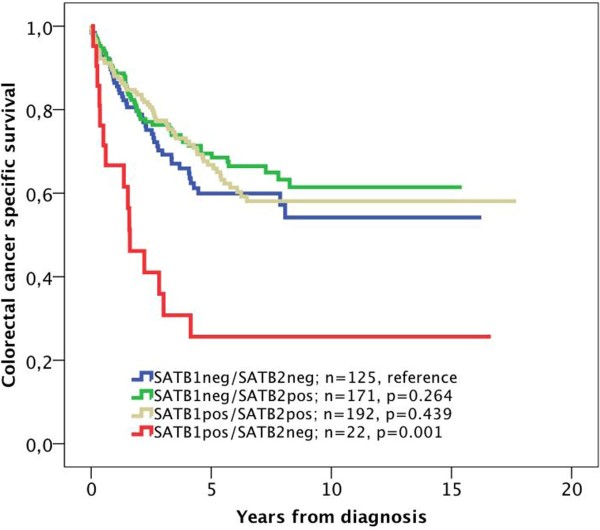
**Kaplan-Meier estimates of the prognostic impact of SATB1 expression in all patients, and patients with SATB2 negative and positive tumours.** Kaplan–Meier estimates of colorectal cancer-specific survival according to combinations of negative and positive SATB1 and SATB2 expression. Log rank P-values correspond to pairwise comparisons of tumours negative for both SATB1 and SATB2 with the other strata.

The significant association of SATB1 expression with MSI status, beta-catenin grades and SATB2 expression led us to examine the modifying effect of these factors on the prognostic value of SATB1 expression. While no such effect was seen for MSI status and beta-catenin overexpression, SATB1 positivity was significantly associated with a shorter CSS in SATB2 negative tumours (p = 0.001, Figure 2B), in contrast to SATB2 positive tumours, where SATB1 was not significantly associated with survival (Figure 2C). Notably, none of the SATB2 negative tumours displayed high SATB1 staining (Figure 3B). The adverse prognosis for SATB1 positive/SATB2 negative tumours was confirmed in univariable Cox regression analysis (HR = 2.63; 95% CI 1.46-4.71, p = 0.001 for CSS and HR = 2.31; 95% CI 1.32-4.04, p = 0.003 for OS), remaining significant in multivariable analysis, adjusted for age, gender, TNM stage and differentiation grade (HR = 2.07; 95% CI 1.06-4.05, p = 0.034 for CSS and HR = 2.05; 95% CI 1.09-3.88, p = 0.026 for OS) (Table 2), with a significant interaction between SATB1 and SATB2 expression (p_interaction_ =0.011 for CSS and 0.015 for OS) (Table 2). The independent prognostic value of SATB1 was lost when vascular invasion was included in the multivariable analysis (data not shown). The prognostic value of SATB2 did not differ according to SATB1 expression (Figure 3).

**Table 2:** Cox proportional hazards analysis of the prognostic interaction of SATB1 with SATB2 expression

	**Cancer specific survival**			**Overall survival**				
	**HR(95%CI)**	***p***		***p†***	**HR(95%CI)**	***p***		***p†***
**All SATB2 positive tumours**		*univariable*				*univariable*		
SATB1 neg	1,00		171(52)		1,00		171(67)	
SATB1 pos	1.09(0.75-1.57)	0.649	192(64)		1.01(0.73-1.41)	0.932	192(77)	
				0.011				0.015
**All SATB2 negative tumours**		*univariable*				*univariable*		
SATB1 neg	1,00		125(46)		1,00		125(56)	
SATB1 pos	2.63(1.46-4.71)	0.001	22(15)		2.31(1.32-4.04)	0.003	22(16)	
		*multivariable*				*multivariable*		
SATB1 neg	1,00		115(40)		1,00		115(48)	
SATB1 pos	2.07(1.06-4.05)	0.034	17(12)		2.05(1.09-3.88)	0.026	17(13)	

Multivariable analysis included adjustment for age (>/<=75 years), gender, T-stage (I-II, III, IV), N-stage (0,1,2), M-stage (0, 1) and differentiation grade (high-intermediate vs low)*. p†:* P-value for term of interaction by Cox multivariate analysis including SATB1 expression, SATB2 expression, and a term of interaction.

## Discussion

The results from this large, prospective cohort study of colorectal cancer demonstrate that SATB1 is expressed in a subset of colorectal carcinomas, and correlates with beta-catenin overexpression, microsatellite stable tumours and SATB2 expression. Furthermore, while not prognostic in the full cohort, SATB1 expression was found to be a factor of poor prognosis in SATB2 negative tumours.

Despite the positive correlation between SATB1 and SATB2 expression in CRC, it is evident that the expression pattern of SATB1 in normal mucosa and CRC differs from SATB2, which is abundantly expressed in a tissue-specific manner in the mucosa of the lower gastrointestinal tract and in the vast majority of CRC [3,4]. Our results demonstrate that normal colorectal mucosa is more or less devoid of SATB1 expression, which is in line with the study by Meng et al. [12]. Furthermore, the proportion of cases with invasive CRC expressing SATB1 was 42% in our study, which is also in line with previous findings by Meng et al. [12], although, in contrast to their study, we found no significant association between SATB1 expression and unfavourable clinicopathological characteristics. Moreover, in contrast to SATB2 [4], the value of SATB1 expression as a predictor of response to adjuvant chemotherapy was not evident.

The antibody used in this study has been validated as being highly specific towards SATB1, and therefore, the risk of a crossreactivity with SATB2 is negligible. This is also supported by the observation that SATB1 was absent in the vast majority of normal colorectal mucosa samples, which is in stark contrast to the strong and abundant expression of SATB2 in non-malignant mucosa of the lower GI-tract [3,4]. Moreover, although the small and quite distinct subset of 18 tumours with a particularly high SATB1 expression (NS = 12) expressed similarly high levels of SATB2, it must be emphasized that the proportion of tumours with high (NS = 12) SATB2 expression was considerably higher, and that the majority of these expressed no or weak levels of SATB1.

The association between SATB1 expression and adverse outcome in SATB2 negative tumours is of potential significance, although based on a post-hoc analysis in a rather small subgroup. This should be considered in future mechanistic studies on the roles and potential interaction of SATB1 and SATB2 in colorectal carcinogenesis and progression, as well as in validatory studies on human CRC samples. In light of the observed synergism between SATB1 and SATB2 at the expression level, their antagonistic impact on prognosis may seem contradictory. Moreover, even if a significant prognostic interaction was observed between SATB1 and SATB2, the prognostic value of SATB2 did not differ by SATB1 expression, which would have been expected if the two proteins were antagonistic. One, more simplistic, explanation for this observation could be that the beneficial prognostic value of SATB2, being expressed in the majority of CRC [3] overrules the potential tumour-promoting effects of SATB1. Another explanation could be that SATB1 and SATB2 exert synergistic effects in the majority of CRC, as reflected in their positive interrelationship, but that, in the absence of SATB2, SATB1 may reprogramme the tumours towards a more malignant phenotype. This hypothesis is partly substantiated by the findings by Wang et al., where SATB1 expression was shown to be down-regulated upon overexpression of SATB2 in a metastatic subclone of the SW480 CRC cell line, and up-regulated after siRNA-mediated silencing of SATB2 in SW480 cells [5]. Moreover, antagonistic activities of SATB1 and SATB2 have been observed in murine embryonic stem cells, where their relative levels appear to regulate the balance of self-renewal versus differentiation [20].

Similar to SATB2 [12], SATB1 expression was associated with microsatellite stable tumours. As regards SATB2, this finding is not unexpected, as an inverse association with MSI has been observed for other markers of colorectal lineage [21], but the role of SATB1 in this respect is less evident and should also be considered in future studies.

Important functions for SATB1 have been implicated in several other cancer forms, and its prognostic value seems to be cancer-type specific [22]. In an initial study on breast cancer, SATB1 was demonstrated to act as a genetic master switch towards a more aggressive tumour behaviour, and moreover, high immunohistochemical expression of SATB1 in tumours fom a large cohort of breast cancer patients was an independent marker of poor prognosis [8]. However, two follow-up studies failed to confirm a role for SATB1 in malignant behaviour of breast cancer cells, and analysis of tumours from several patient cohorts revealed no association between high SATB1 mRNA expression and adverse outcome [23,24]. Possibly, the prognostic value of SATB1 in breast cancer may depend on hormone receptor status [24-26]. In gastric cancer, SATB1 expression has been reported to correlate with a more malignant phenotype and poor prognosis whereas in squamous cell carcinoma of the lung, downregulation of SATB1 was demonstrated to be associated with an impaired survival [27].

Notably, observations of a prognostic disconcordance between mRNA and protein levels of candidate biomarkers are not uncommon, and from a clinical viewpoint, immunohistochemistry has several advantages compared to gene expression analyses, since it allows for quantitative assessment of proteins in a morphological context, which might have important prognostic implications. Moreover, SATB1 is not only expressed in tumour cells, but also in stromal lymphocytes, and, importantly, the prognostic impact was evident even at low levels of expression, not least in the subgroup of SATB2 negative tumours. These findings are consistent with the study by Han et al., where the majority of the breast cancer samples were denoted as having weak immunohistochemical expression of SATB1, which still had considerable prognostic impact [8]. It should also be pointed out that in the study by Meng et al., there was a significant correlation of SATB1 mRNA levels and protein expression, both of which were associated with unfavourable clinicopathological characteristics [12].

## Conclusion

The results of this study demonstrate that SATB1 expression in colorectal cancer is significantly associated with beta-catenin overexpression, microsatellite stability and SATB2 expression. Furthermore, while not prognostic in unstratified analysis, SATB1 expression is shown to be a factor of poor prognosis in SATB2 negative tumours. Future studies are needed to elucidate the potential mechanisms by which SATB1 affects colorectal cancer progression, not least its potential synergistic or antagonostic effects with SATB2.

## Abbreviations

SATB1: Special AT-rich sequence-binding protein 1; SATB2: Special AT-rich sequence-binding protein 2; CRC: Colorectal cancer; CSS: Cancer-specific survival; OS: Overall survival; MSI: Microsatellite instability; MSS: Microsatellite stability.

## Competing interests

The authors declare that no competing interests exist.

## Authors' contributions

BN carried out all IHC stainings and evaluation, and drafted the manuscript. HJ carried out the ELISA and WB experiments and helped draft the manuscript. SW collected clinical data and participated in the evaluation of the immunohistochemistry. DO’C assisted with the antibody validation. KEL participated in the evaluation of the immunohistochemistry. MU contributed to the conception and design of the study. KJ conceived of the study, carried out the histopathological re-evaluation, evaluated the immunohistochemistry, and drafted the manuscript. JE assisted with the collection of clinical data, and drafted the manuscript. All authors read and approved the final manuscript.

## Supplementary Material

Additional file 1 **Sample immunohistochemical images of beta-catenin grades.** Sample images of beta-catenin staining representing (A) normal colorectal epithelial cells with intact membranous beta-catenin expression, and colorectal cancers with (B) intact membranous expression (grade 1), (C) positive cytoplasmic expression (grade 2), and (D) positive nuclear and cytoplasmic expression (grade 3). Click here for file

Additional file 2 **Validation of the anti-SATB1 antibody.** The specificity of the anti-SATB1 antibody was analysed using (A) ELISA and (B) Western blot against purified recombinant SATB1 and SATB2 proteins. An anti-SATB2 antibody was included as a control in the western blot experiment, which shows that both antibodies are specific for their respective proteins. Click here for file

Additional file 3 **Distribution of SATB1 staining in the full cohort.** Distribution of all nuclear scores (fraction x intensity) of SATB1 expression in the full cohort. Percentage is shown on the y-axis and absolute numbers above the bars. Click here for file
