# Supplementary figures and images for: Molecular correlates and prognostic significance of SATB1 expression in colorectal cancer
Source: Diagn Pathol. 2012 Aug 30;7:115. doi: 10.1186/1746-1596-7-115 (PMC3523011; doi:10.1186/1746-1596-7-115)

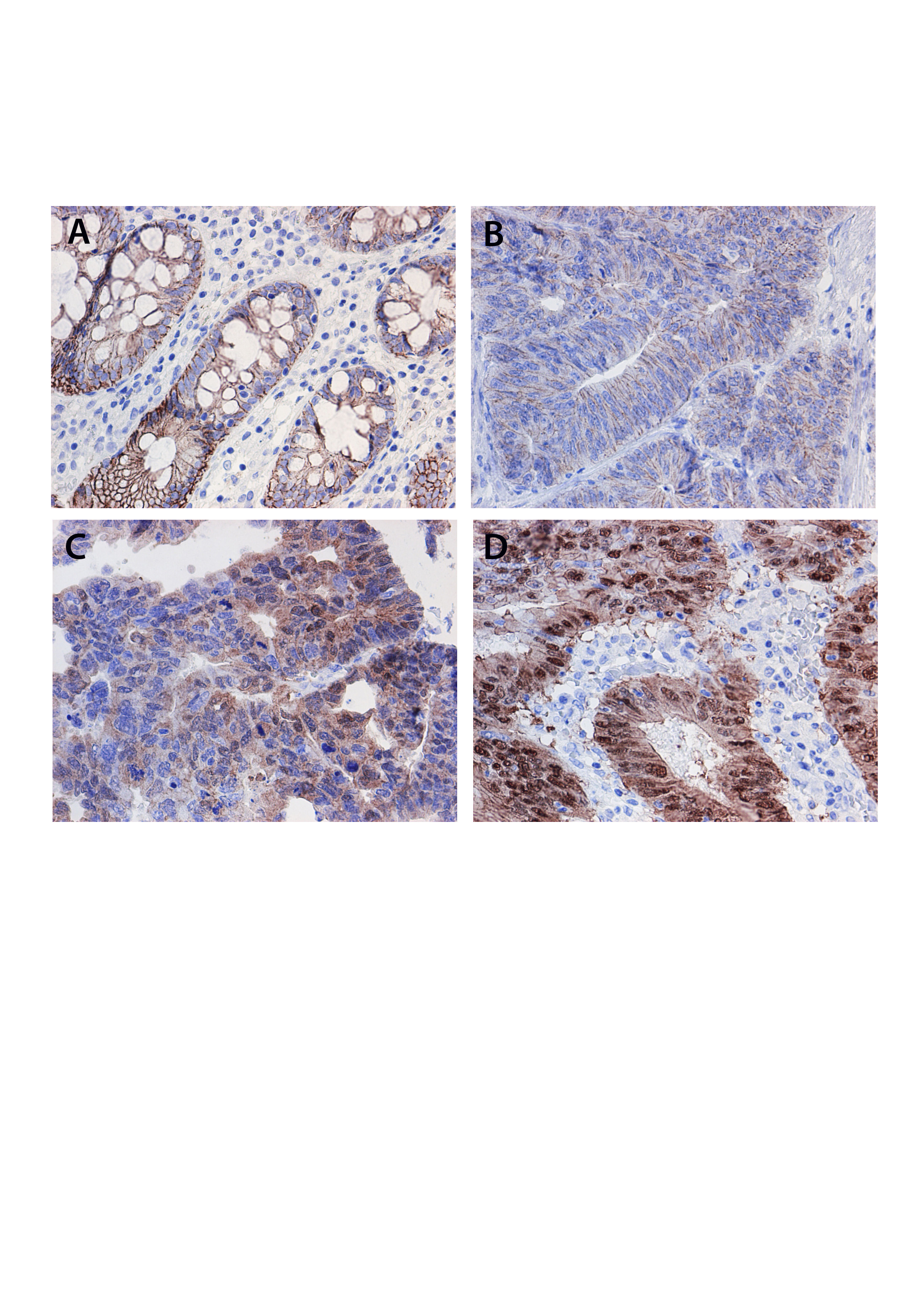

Supplement: Additional file 1 — Sample immunohistochemical images of beta-catenin grades. Sample images of beta-catenin staining representing (A) normal colorectal epithelial cells with intact membranous beta-catenin expression, and colorectal cancers with (B) intact membranous expression (grade 1), (C) positive cytoplasmic expression (grade 2), and (D) positive nuclear and cytoplasmic expression (grade 3). [file 1746-1596-7-115-S1.jpeg]

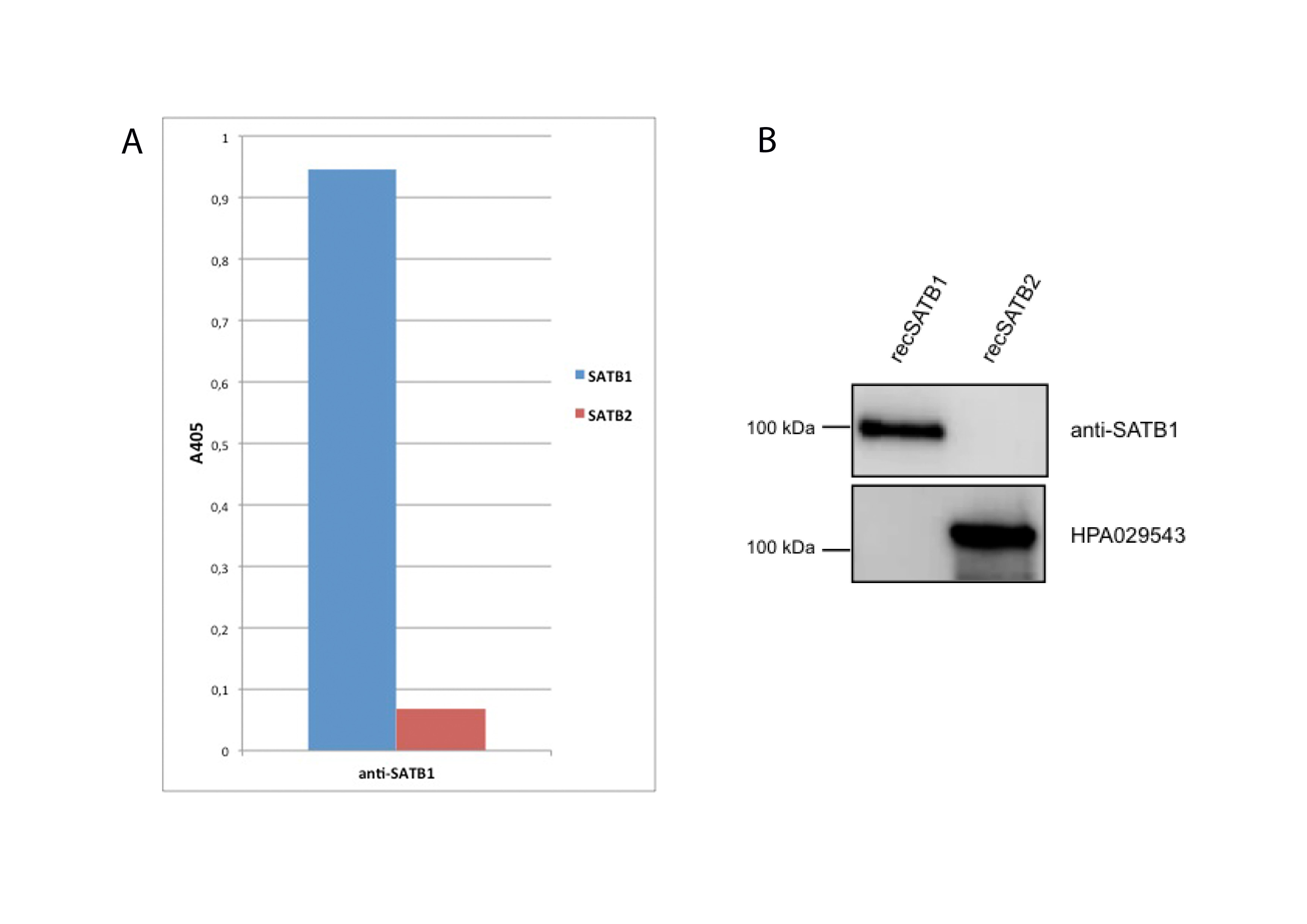

Supplement: Additional file 2 — Validation of the anti-SATB1 antibody. The specificity of the anti-SATB1 antibody was analysed using (A) ELISA and (B) Western blot against purified recombinant SATB1 and SATB2 proteins. An anti-SATB2 antibody was included as a control in the western blot experiment, which shows that both antibodies are specific for their respective proteins. [file 1746-1596-7-115-S2.jpeg]

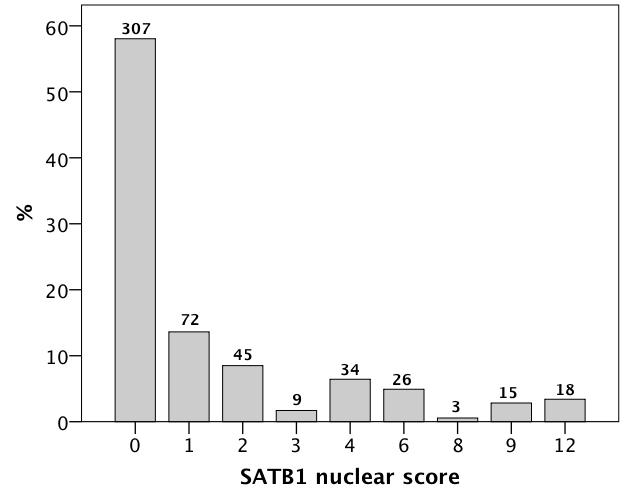

Supplement: Additional file 3 — Distribution of SATB1 staining in the full cohort. Distribution of all nuclear scores (fraction x intensity) of SATB1 expression in the full cohort. Percentage is shown on the y-axis and absolute numbers above the bars. [file 1746-1596-7-115-S3.jpeg]
